# Supplementary material for: Mutational biases favor complexity increases in protein interaction networks after gene duplication
Source: Mol Syst Biol. 2024 Mar 18;20(5):549–72. doi: 10.1038/s44320-024-00030-z (PMC11066126; doi:10.1038/s44320-024-00030-z)
Supplement: Supplementary file 2 — Appendix [file 44320_2024_30_MOESM2_ESM.pdf]

# Appendix

## Mutational biases promote neutral increases in the complexity of protein interaction networks following gene duplication

Angel F. Cisneros, Lou Nielly-Thibault, Saurav Mallik, Emmanuel D. Levy, Christian R. Landry

Table of contents:

Page
- Appendix note 1..........2
- Appendix note 2..........5
- Appendix Supplementary figures..........13
    - Appendix Figure S1..........14
    - Appendix Figure S2..........15
    - Appendix Figure S3..........16
    - Appendix Figure S4..........17
    - Appendix Figure S5..........18
    - Appendix Figure S6..........19
    - Appendix Figure S7..........20
    - Appendix Figure S8..........21
    - Appendix Figure S9..........22
    - Appendix Figure S10..........23

# Appendix Note 1

Two molecules of A form the dimer AA. Concentrations are at a steady state determined by the law of mass action according to this reaction network :

| | | |
|---|---|---|
| =>sA | A | =>dA |
| 2A | r1<=>r2 | AA |
| | AA | =>dAA |

where $s_A$ is the synthesis rate of A (units of concentration*time^-1), $r_1$ is the dissociation rate of AA (units of concentration^-1*time^-1), $r_2$ is the association rate of A, and $d_A, d_{AA}$ are degradation rates (units of time^-1).

Let cA, cAA denote the concentrations of the two molecular species :

Because at equilibrium the concentration of A is constant :

**Equation 1 :** $s_A + 2 r_1 c_{AA} = d_A c_A + 2 r_2 c_A^2$

Because at equilibrium the concentration of AA is constant :

**Equation 2 :** $r_2 c_A^2 = d_{AA} c_{AA} + r_1 c_{AA}$

Starting from equation 2, we get an expression for $c_{AA}$ :

$$r_2 c_A^2 = d_{AA} c_{AA} + r_1 c_{AA}$$

$$0 = (d_{AA} + r_1) c_{AA} - r_2 c_A^2$$

$$c_{AA} = \frac{r_2 c_A^2}{d_{AA} + r_1}$$

With $k_{AA}$ defined as $\frac{r_2}{d_{AA} + r_1}$ :

$$c_{AA} = k_{AA} c_A^2$$

Starting from equation 1 :

$$s_A + 2 r_1 c_{AA} = d_A c_A + 2 r_2 c_A^2$$

$$0 = 2 r_2 c_A^2 + d_A c_A - s_A - 2 r_1 c_{AA}$$

By substituting $c_{AA}$:

$$0 = 2 r_2 c_A^2 + d_A c_A - s_A - 2 r_1 k_{AA} c_A^2$$

From the definition $k_{AA}=\frac{r_2}{d_{AA}+r_1}$ , we get $r_2-r_1 k_{AA}=d_{AA} k_{AA}$ and therefore :

$$0=2r_2 c_A^2+d_A c_A-s_A-2r_1 c_A^2 k_{AA}$$

$$0=2r_2 c_A^2+d_A c_A-s_A-2r_1 c_A^2 k_{AA}$$

$$0=2c_A^2\left(d_{AA} k_{AA}\right)+d_A c_A-s_A$$

Applying the quadratic formula :

$$c_A=\frac{-b\pm\sqrt{b^2-4ac}}{2a}=\frac{-d_A\pm\sqrt{d_A^2-4\left(2d_{AA}k_{AA}\right)\left(-s_A\right)}}{2\left(2d_{AA}\right)\left(k_{AA}\right)}$$

$$c_A=\frac{-d_A\pm\sqrt{d_A^2+8d_{AA}k_{AA}s_A}}{4d_{AA}k_{AA}}$$

In the formula above, the coefficients are such that $a>0$ , $b\geq 0$ , $c\leq 0$ and thus $b\leq\sqrt{b^2-4ac}$ . It follows that $-b+\sqrt{b^2-4ac}$ is non-negative, while $-b-\sqrt{b^2-4ac}$ is non-positive. We therefore choose $-b+\sqrt{b^2-4ac}$ to obtain a non-negative value of $c_B$ .

$$c_A=\frac{-d_A+\sqrt{d_A^2+8d_{AA}k_{AA}s_A}}{4d_{AA}k_{AA}}$$

# Appendix Note 2

Two molecules $A$ and $B$ form the dimers $AA$ , $AB$ and $BB$ . Concentrations are at a steady state determined by the law of mass action according to this reaction network:

=>sA **A** =>dA

=>sB **B** =>dB

**2A** r1<=>r2 **AA**

**2B** r3<=>r4 **BB**

**A + B** r5<=>r6 **AB**

**AA** =>dAA

**BB** =>dBB

**AB** =>dAB

where $s_A, s_B$ are synthesis rates (units of concentration * time^-1), $r_1, r_3, r_5$ are dissociation rates (units of time^-1), $r_2, r_4, r_6$ are association rates (units of concentration^-1 * time^-1), and $d_A, d_{AA}, d_{AB}, d_{BA}, d_{BB}, d_B$ are degradation rates (units of time^-1).

Let $c_A, c_{AA}, c_{AB}, c_{BB}, c_B$ denote the concentrations of the five molecular species.

Because the concentration of $A$ is constant:
**Equation 1:** $s_A + 2r_1 c_{AA} + r_5 c_{AB} = d_A c_A + 2r_2 c_A^2 + r_6 c_A c_B$

Because the concentration of $AA$ is constant:
**Equation 2:** $r_2 c_A^2 = d_{AA} c_{AA} + r_1 c_{AA}$

Because the concentration of $AB$ is constant:
**Equation 3:** $r_6 c_A c_B = d_{AB} c_{AB} + r_5 c_{AB}$

**A/B symmetry**: The reaction network is symmetric with respect to the two lists of variables $[c_A, c_{AA}, s_A, d_A, r_1, r_2, d_{AA}]$ and $[c_B, c_{BB}, s_B, d_B, r_3, r_4, d_{BB}]$ . This means that any equation will remain true if we simultaneously replace each of these parameters by the corresponding parameter from the other list.

Starting from equation 1, we can get an expression for $c_{AA}$ :

$$s_A + 2r_1 c_{AA} + r_5 c_{AB} = d_A c_A + 2r_2 c_A^2 + r_6 c_A c_B$$

$$c_{AA} = \frac{d_A c_A + 2 r_2 c_A^2 + r_6 c_A c_B - s_A - r_5 c_{AB}}{2 r_1}$$

Starting from equation 2, we can get another expression for $c_{AA}$ :

$$r_2 c_A^2 = d_{AA} c_{AA} + r_1 c_{AA}$$

$$c_{AA} = \frac{r_2}{d_{AA} + r_1} c_A^2$$

With $k_{AA}$ defined as $\frac{r_2}{d_{AA} + r_1}$ :

$$c_{AA} = k_{AA} c_A^2$$

By equating these two expressions of $c_{AA}$ , we can get an expression for $c_{AB}$ :

$$k_{AA} c_A^2 = \frac{d_A c_A + 2 r_2 c_A^2 + r_6 c_A c_B - s_A - r_5 c_{AB}}{2 r_1}$$

$$2 r_1 k_{AA} c_A^2 = d_A c_A + 2 r_2 c_A^2 + r_6 c_A c_B - s_A - r_5 c_{AB}$$

$$r_5 c_{AB} = d_A c_A + 2 r_2 c_A^2 + r_6 c_A c_B - s_A - 2 r_1 k_{AA} c_A^2$$

$$r_5 c_{AB} = d_A c_A + 2(r_2 - r_1 k_{AA}) c_A^2 + r_6 c_A c_B - s_A$$

$$c_{AB} = \frac{d_A c_A + 2(r_2 - r_1 k_{AA}) c_A^2 + r_6 c_A c_B - s_A}{r_5}$$

From the definition $k_{AA} = \frac{r_2}{d_{AA} + r_1}$ , we get $r_2 - r_1 k_{AA} = d_{AA} k_{AA}$ and therefore:

$$c_{AB} = \frac{d_A c_A + 2 d_{AA} k_{AA} c_A^2 + r_6 c_A c_B - s_A}{r_5}$$

Starting from equation 3, we can get another expression for $c_{AB}$ :

$$r_6 c_A c_B = d_{AB} c_{AB} + r_5 c_{AB}$$

$$c_{AB} = \frac{r_6}{d_{AB} + r_5} c_A c_B$$

With $k_{AB}$ defined as $\frac{r_6}{d_{AB} + r_5}$ :

$$c_{AB} = k_{AB} c_A c_B$$

By equating these two values of $c_{AB}$ , we can get an expression for $c_B$ :

$$k_{AB} c_A c_B = \frac{d_A c_A + 2 d_{AA} k_{AA} c_A^2 + r_6 c_A c_B - s_A}{r_5}$$

$$r_5 k_{AB} c_A c_B = d_A c_A + 2 d_{AA} k_{AA} c_A^2 + r_6 c_A c_B - s_A$$

$$(r_5 k_{AB} - r_6) c_A c_B = d_A c_A + 2 d_{AA} k_{AA} c_A^2 - s_A$$

$$c_B = \frac{d_A c_A + 2 d_{AA} k_{AA} c_A^2 - s_A}{(r_5 k_{AB} - r_6) c_A}$$

From the definition $k_{AA} = \frac{r_6}{d_{AB} + r_5}$ , we get $r_6 - r_5 k_{AB} = d_{AB} k_{AB}$ and therefore:

$$c_B = \frac{d_A c_A + 2 d_{AA} k_{AA} c_A^2 - s_A}{-d_{AB} k_{AB} c_A}$$

By applying the A/B symmetry to this equation, we can get another expression for $c_B$ :

$$c_A = \frac{d_B c_B + 2 d_{BB} k_{BB} c_B^2 - s_B}{-d_{AB} k_{AB} c_B}$$

where $k_{BB}$ is defined as $\frac{r_4}{d_{BB} + r_3}$

$$-d_{AB} k_{AB} c_B c_A = d_B c_B + 2 d_{BB} k_{BB} c_B^2 - s_B$$

$$0 = (d_B + d_{AB} k_{AB} c_A) c_B + 2 d_{BB} k_{BB} c_B^2 - s_B$$

$$0 = 2 d_{BB} k_{BB} c_B^2 + (d_B + d_{AB} k_{AB} c_A) c_B - s_B$$

Applying the quadratic formula:

$$c_B = \frac{-b \pm \sqrt{b^2 - 4ac}}{2a} = \frac{-(d_B + d_{AB} k_{AB} c_A) \pm \sqrt{(d_B + d_{AB} k_{AB} c_A)^2 + 8 d_{BB} k_{BB} s_B}}{4 d_{BB} k_{BB}}$$

In the formula above, the coefficients are such that $a > 0$ , $b \geq 0$ , $c \leq 0$ and thus $b \leq \sqrt{b^2 - 4ac}$ . It follows that $-b + \sqrt{b^2 - 4ac}$ is non-negative, while $-b - \sqrt{b^2 - 4ac}$ is non-positive. We therefore choose $-b + \sqrt{b^2 - 4ac}$ to obtain a non-negative value of $c_B$ .

$$c_B = \frac{-(d_B + d_{AB} k_{AB} c_A) + \sqrt{(d_B + d_{AB} k_{AB} c_A)^2 + 8 d_{BB} k_{BB} s_B}}{4 d_{BB} k_{BB}}$$

By equating the two expressions that we obtained for $c_B$, we get:

$$\frac{d_A c_A + 2 d_{AA} k_{AA} c_A^2 - s_A}{-d_{AB} k_{AB} c_A} = \frac{-(d_B + d_{AB} k_{AB} c_A) + \sqrt{(d_B + d_{AB} k_{AB} c_A)^2 + 8 d_{BB} k_{BB} s_B}}{4 d_{BB} k_{BB}}$$

$$\frac{s_A - d_A c_A - 2 d_{AA} k_{AA} c_A^2}{d_{AB} k_{AB} c_A} = \frac{-(d_B + d_{AB} k_{AB} c_A) + \sqrt{(d_B + d_{AB} k_{AB} c_A)^2 + 8 d_{BB} k_{BB} s_B}}{4 d_{BB} k_{BB}}$$

Taking $x = d_{AA} k_{AA}$ , $y = d_{BB} k_{BB}$ and $z = d_{AB} k_{AB}$ , we obtain:

$$\frac{s_A - d_A c_A - 2 x c_A^2}{z c_A} = \frac{-(d_B + z c_A) + \sqrt{(d_B + z c_A)^2 + 8 y s_B}}{4 y}$$

$$4 y s_A - 4 y d_A c_A - 8 y x c_A^2 = -d_B z c_A - z^2 c_A^2 + \sqrt{(d_B z c_A + z^2 c_A^2)^2 + 8 y s_B z^2 c_A^2}$$

$$(d_B z c_A + z^2 c_A^2) + (4 y s_A - 4 y d_A c_A - 8 y x c_A^2) = \sqrt{(d_B z c_A + z^2 c_A^2)^2 + 8 y s_B z^2 c_A^2}$$

$$\left((d_B z c_A + z^2 c_A^2) + (4 y s_A - 4 y d_A c_A - 8 y x c_A^2)\right)^2 = (d_B z c_A + z^2 c_A^2)^2 + 8 y s_B z^2 c_A^2$$

$$2(d_B z c_A + z^2 c_A^2)(4 y s_A - 4 y d_A c_A - 8 y x c_A^2) + (4 y s_A - 4 y d_A c_A - 8 y x c_A^2)^2 = 8 y s_B z^2 c_A^2$$

$$8d_B s_A y z c_A - 8d_A d_B y z c_A^2 - 16 d_B x y z c_A^3 + 8 s_A y z^2 c_A^2 - 8 d_A y z^2 c_A^3 - 16 x y z^2 c_A^4$$
$$+$$
$$16 s_A^2 y^2 - 16 s_A d_A y^2 c_A - 32 s_A x y^2 c_A^2 - 16 s_A d_A y^2 c_A + 16 d_A^2 y^2 c_A^2 + 32 d_A x y^2 c_A^3$$
$$- 32 s_A x y^2 c_A^2 + 32 d_A x y^2 c_A^3 + 64 x^2 y^2 c_A^4$$
$$=$$
$$8 y s_B z^2 c_A^2$$

$$8d_B s_A y z c_A - 8d_A d_B y z c_A^2 - 16 d_B x y z c_A^3 + 8 s_A y z^2 c_A^2 - 8 d_A y z^2 c_A^3 - 16 x y z^2 c_A^4$$
$$+$$
$$16 s_A^2 y^2 - 16 s_A d_A y^2 c_A - 32 s_A x y^2 c_A^2 - 16 s_A d_A y^2 c_A + 16 d_A^2 y^2 c_A^2 + 32 d_A x y^2 c_A^3$$
$$- 32 s_A x y^2 c_A^2 + 32 d_A x y^2 c_A^3 + 64 x^2 y^2 c_A^4 - 8 y s_B z^2 c_A^2$$
$$=$$
$$0$$

Dividing by $y$ :

$$8d_B s_A z c_A - 8d_A d_B z c_A^2 - 16 d_B x z c_A^3 + 8 s_A z^2 c_A^2 - 8 d_A z^2 c_A^3 - 16 x z^2 c_A^4$$
$$+$$
$$16 s_A^2 y - 16 s_A d_A y c_A - 32 s_A x y c_A^2 - 16 s_A d_A y c_A + 16 d_A^2 y c_A^2 + 32 d_A x y c_A^3$$
$$- 32 s_A x y c_A^2 + 32 d_A x y c_A^3 + 64 x^2 y c_A^4 - 8 s_B z^2 c_A^2$$
$$=$$
$$0$$

Dividing by 8:

$$d_B s_A z c_A - d_A d_B z c_A^2 - 2 d_B x z c_A^3 + s_A z^2 c_A^2 - d_A z^2 c_A^3 - 2 x z^2 c_A^4$$
$$+$$
$$2 s_A^2 y - 2 s_A d_A y c_A - 4 s_A x y c_A^2 - 2 s_A d_A y c_A + 2 d_A^2 y c_A^2 + 4 d_A x y c_A^3$$
$$- 4 s_A x y c_A^2 + 4 d_A x y c_A^3 + 8 x^2 y c_A^4 - s_B z^2 c_A^2$$
$$=$$
$$0$$

$$0 = (8x^2 y - 2xz^2)c_A^4 + (8d_A xy - 2d_B xz - d_A z^2)c_A^3$$
$$+ (s_A z^2 - d_A d_B z - 8s_A xy + 2d_A^2 y - s_B z^2)c_A^2 + (s_A d_B z - 4s_A d_A y)c_A + 2s_A^2 y$$

$$0 = 2x(4xy - z^2)c_A^4 + (d_A(8xy - z^2) - 2d_B xz)c_A^3$$
$$+ (2yd_A^2 + s_A(z^2 - 8xy) - d_A d_B z - z^2 s_B)c_A^2 + s_A(d_B z - 4d_A y)c_A + 2s_A^2 y$$

Dividing by $z$ :

$$0 = 2x\left(4\frac{xy}{z} - z\right)c_A^4 + \left(d_A\left(8\frac{xy}{z} - z\right) - 2d_B x\right)c_A^3$$
$$+ \left(\frac{2yd_A^2}{z} + s_A\left(z - 8\frac{xy}{z}\right) - d_A d_B - zs_B\right)c_A^2 + s_A\left(d_B - 4d_A\frac{y}{z}\right)c_A + 2s_A^2\frac{y}{z}$$

Eliminating $x$ , $y$ and $z$ using their definitions $x = d_{AA}k_{AA}$ , $y = d_{BB}k_{BB}$ and $z = d_{AB}k_{AB}$ , we obtain:

$$0 = 2d_{AA}k_{AA}\left(4\frac{d_{AA}k_{AA}d_{BB}k_{BB}}{d_{AB}k_{AB}} - d_{AB}k_{AB}\right)c_A^4$$
$$+ \left(d_A\left(8\frac{d_{AA}k_{AA}d_{BB}k_{BB}}{d_{AB}k_{AB}} - d_{AB}k_{AB}\right) - 2d_B d_{AA}k_{AA}\right)c_A^3$$
$$+ \left(\frac{2d_{BB}k_{BB}d_A^2}{d_{AB}k_{AB}} + s_A\left(d_{AB}k_{AB} - 8\frac{d_{AA}k_{AA}d_{BB}k_{BB}}{d_{AB}k_{AB}}\right) - d_A d_B - d_{AB}k_{AB}s_B\right)c_A^2$$
$$+ s_A\left(d_B - 4d_A\frac{d_{BB}k_{BB}}{d_{AB}k_{AB}}\right)c_A$$
$$+ 2s_A^2\frac{d_{BB}k_{BB}}{d_{AB}k_{AB}}$$

This formula can be used to compute the values of the coefficients of the 4th degree polynomial given the values of the chemical parameters. The resulting values of the coefficients can then be used by a numerical solver to compute the 4 possible values of $c_A$ (the 4 roots of the polynomial). The

corresponding steady-state concentrations of the other molecular species can then be computed using the formulas derived above.

$$c_B = \frac{d_A c_A + 2 d_{AA} k_{AA} c_A^2 - s_A}{-d_{AB} k_{AB} c_A} = \frac{s_A - 2 d_{AA} k_{AA} c_A^2 - d_A c_A}{d_{AB} k_{AB} c_A} = \frac{s_A / c_A - 2 d_{AA} k_{AA} c_A - d_A}{d_{AB} k_{AB}}$$

$$c_{AA} = k_{AA} c_A^2 = \frac{r_2}{d_{AA} + r_1} c_A^2$$

$$c_{BB} = k_{BB} c_B^2 = \frac{r_4}{d_{BB} + r_3} c_B^2$$

$$c_{AB} = k_{AB} c_A c_B = \frac{r_6}{d_{AB} + r_5} c_A c_B$$

Among the 4 possible values of $c_A$, the one that results in positive values for the concentrations of all five molecular species is the physically correct solution.

# Supplementary Figures

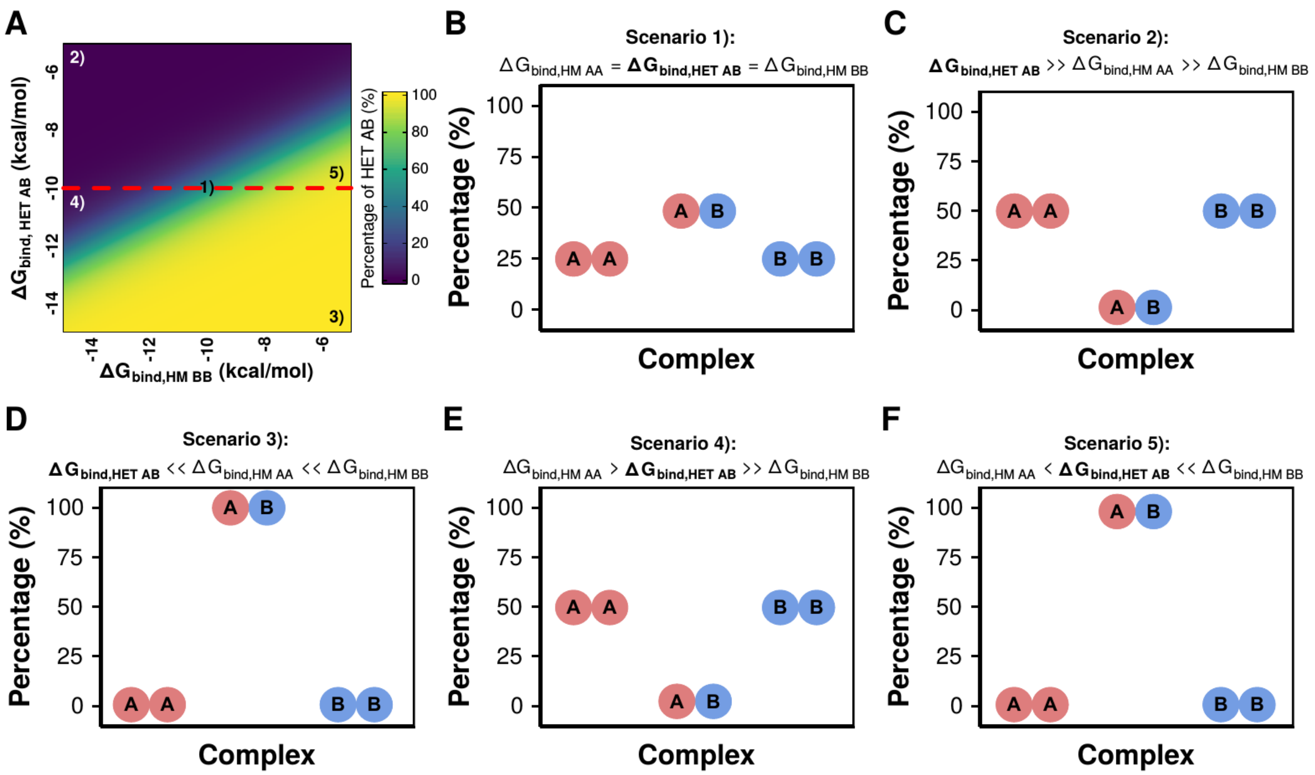


**Appendix Figure S1. The equilibrium between homodimers and heterodimers is determined by binding affinities.**

**A.** Effect of variation in $\Delta G_{bind,AB}$ and $\Delta G_{bind,BB}$ on the percentage of heterodimers, similar to Figure 1G. The dashed red line indicates the value of $\Delta G_{bind,AA}$, which is kept constant at -10 kcal/mol. Numbered labels inside the heatmap indicate the different scenarios described in the rest of the panels.

**B.** Scenario 1): Directly after the duplication, the binding affinities of the three dimers are identical, leading to a 25% AA:50% AB:25% BB equilibrium.

**C.** Scenario 2): Homomers dominate when the heterodimer has the weakest binding affinity.

**D.** Scenario 3): Heterodimers dominate when they have the strongest binding affinity.

**E.** Scenario 4): Homomers dominate when the binding affinity of the heterodimer is slightly stronger than that of one of the homodimers but much weaker than the other one.

**F.** Scenario 5): Heterodimers dominate when the binding affinity of the heterodimer is slightly weaker than that of one of the homodimers but much stronger than the other one.

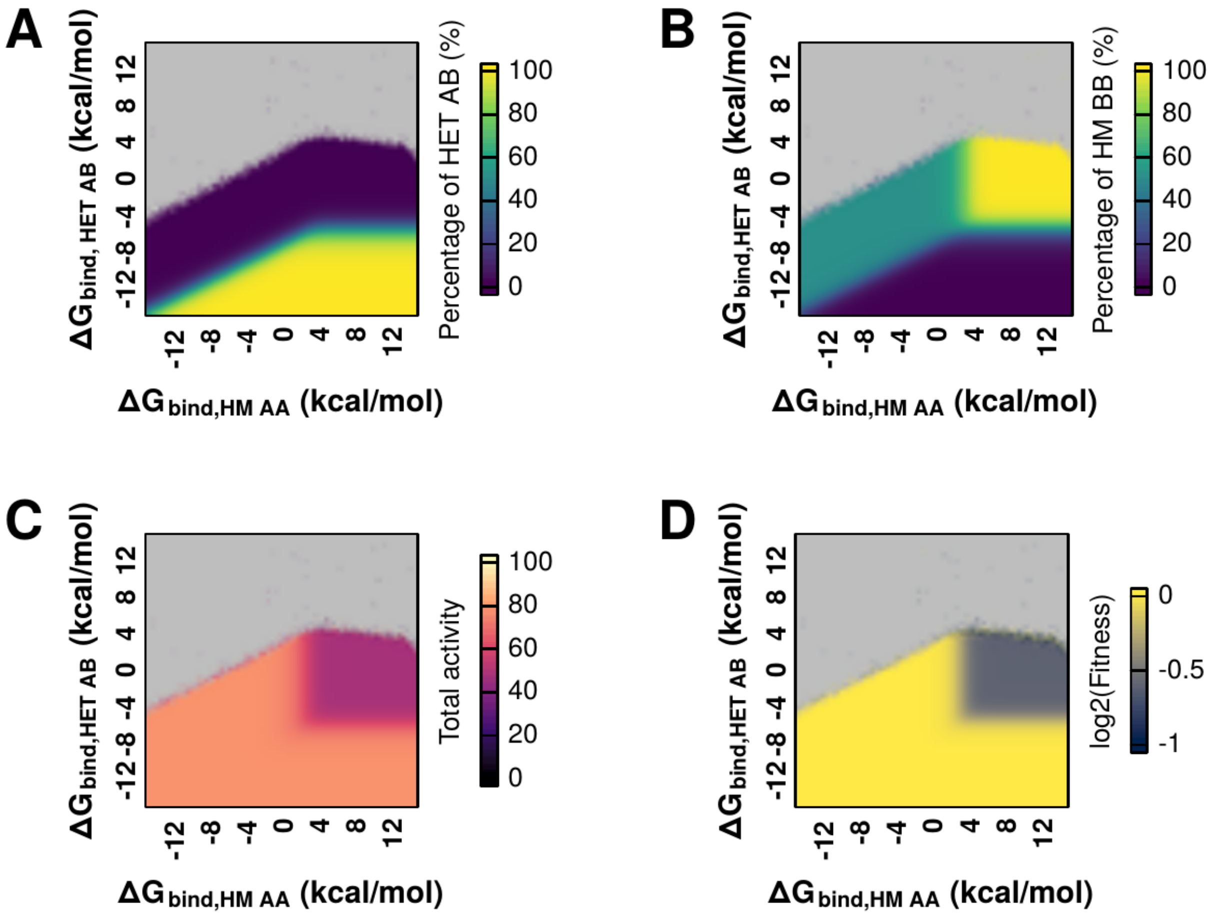


**Appendix Figure S2. Effects of an extended range of different values of binding affinity ($\Delta G_{bind,AB}$ and $\Delta G_{bind,AA}$) on the equilibrium between homo- and heterodimers.**

**A-D.** Effect of variation in $\Delta G_{bind,HET\ AB}$ and $\Delta G_{bind,HM\ AA}$ on the percentage of the heterodimer (HET AB) (A), percentage of one of the homodimers (HM AA) (B), and the total activity (C), and fitness (D) of the system. For panels A-D, $\Delta G_{bind,HM\ BB}$ was kept constant at -10 kcal /mol. The space in grey at the top of each heatmap represents the sets of values for which the equation solver could not find an equilibrium, because the polynomial coefficients (proportional to $(k_{AA}^2 * k_{BB})/k_{AB}$, see equations 2 and 5 in Methods) become too large.

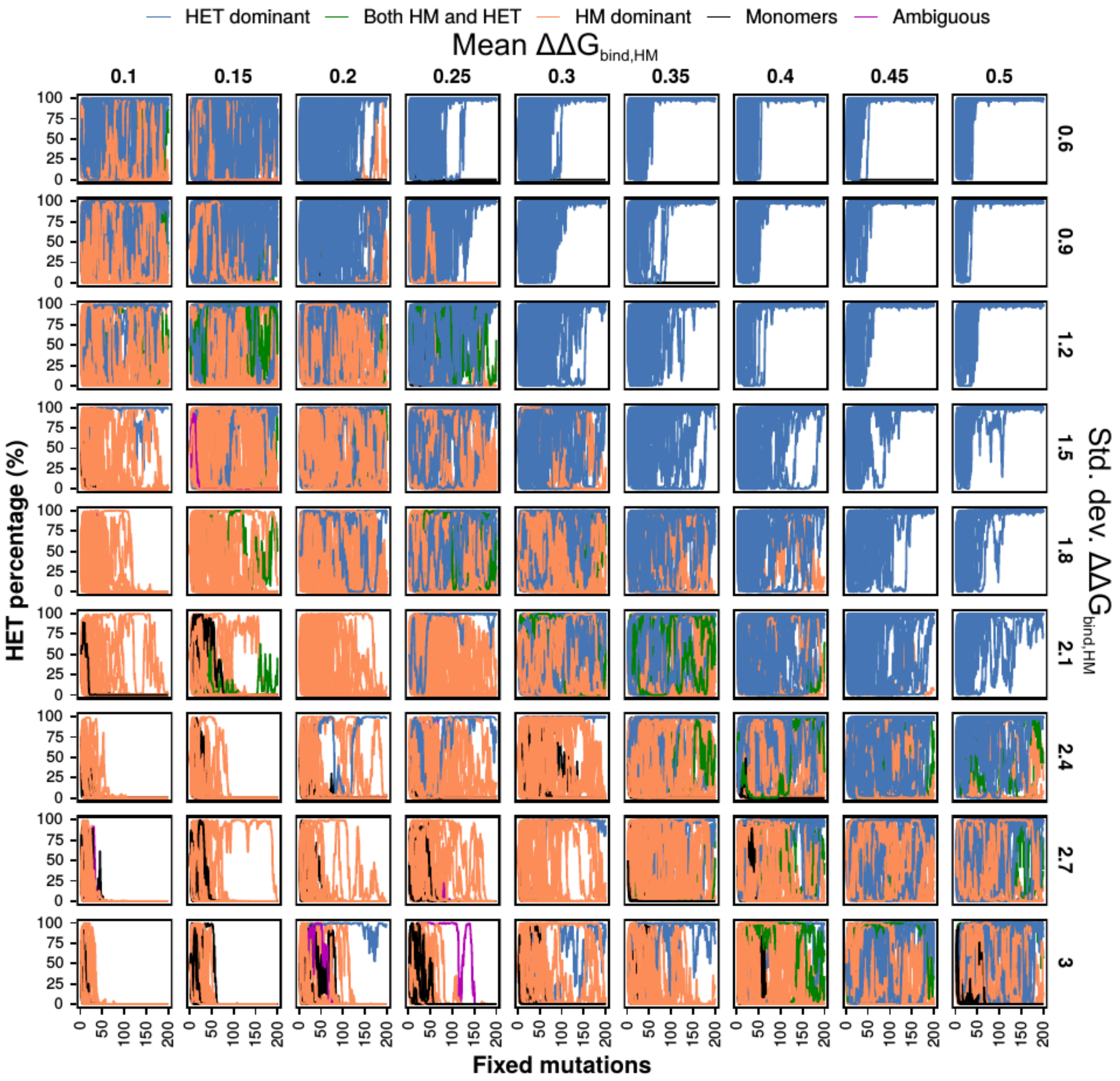


**Appendix Figure S3. Trajectories of individual replicates of parametric simulations.** Panels show trajectories of 50 individual replicates of parametric simulations with different means and standard deviations for the distribution of effects on $\Delta G_{bind,HM}$ (same data as in figure 2B). Replicates were colored according to the equilibrium concentrations of complexes at the end of each simulation following the classification outlined in section 4 of the methods: HET dominant: 70 <= pAB, HM dominant: 70 <= (pAA + pBB), both HM and HET: 70 <= (pAB + pAA + pBB) AND 70 >= pAB AND 70 >= (pAA + pBB), monomers: 70 <= (pA + pB), ambiguous: 70 >= (pAB + pAA + pBB) AND 70 >= (pA + pB).

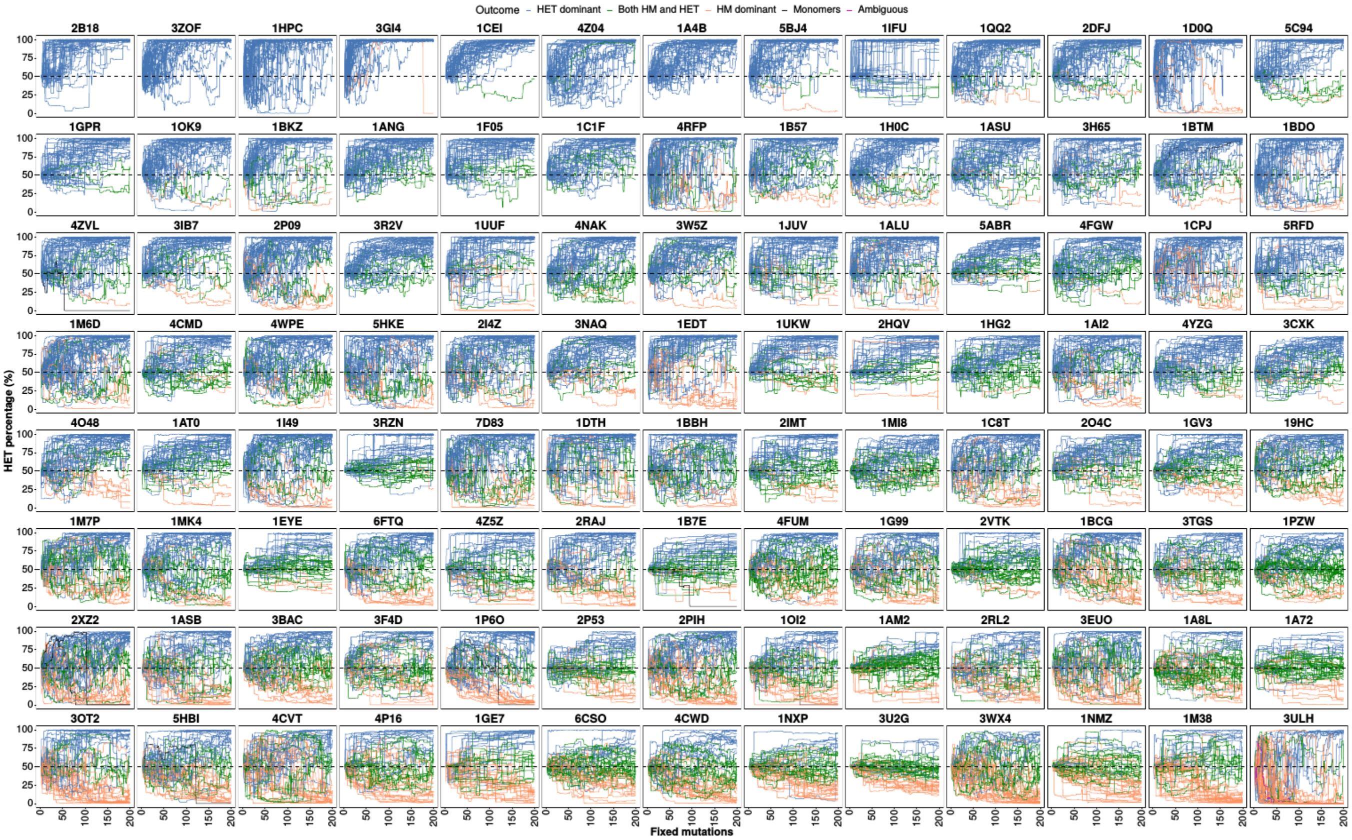


**Appendix Figure S4. Trajectories of individual replicates of simulations with structures.** Panels show trajectories of 50 individual replicates of simulations using the distributions of mutational effects estimated for each PDB structure (same data as in figure 2C). Replicates were colored according to the equilibrium concentrations of complexes at the end of each simulation following the classification outlined in section 4 of the methods: HET dominant: 70 <= pAB, HM dominant: 70 <= (pAA + pBB), both HM and HET: 70 <= (pAB + pAA + pBB) AND 70 >= pAB AND 70 >= (pAA + pBB), monomers: 70 <= (pA + pB), ambiguous: 70 >= (pAB + pAA + pBB) AND 70 >= (pA + pB).

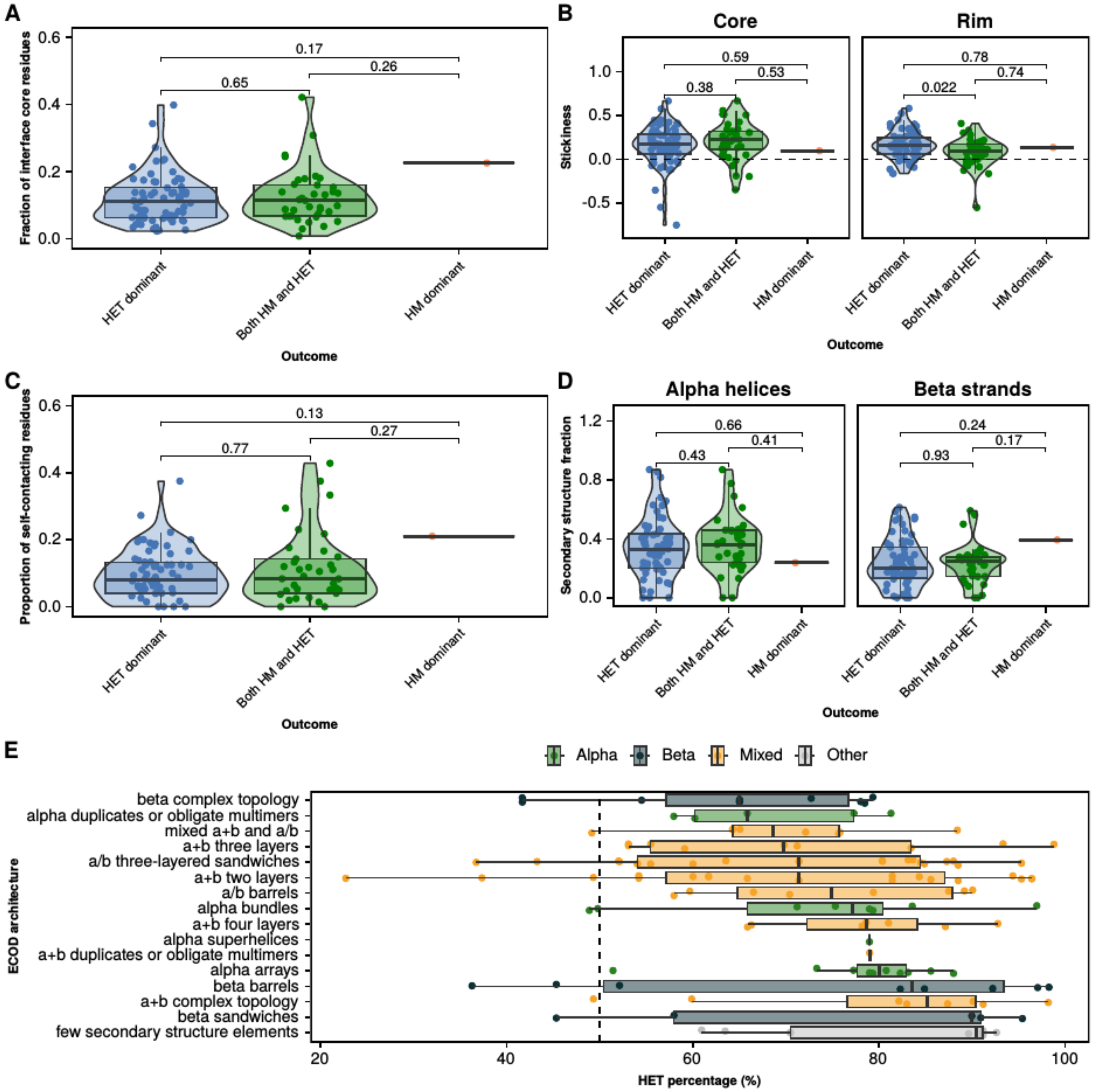


**Appendix Figure S5. No clear effects of structural properties of protein interaction interfaces on the observed outcomes.**

**A-D.** Distributions for all 104 structures tested for fraction of residues of the whole protein that are located in the interface core (A), the average stickiness of the interface core and rim (B), the proportion of residues in the interface core and rim that are within 4 Angstroms of their counterparts in the opposite subunit (C), and the percentage of alpha helices and beta strands at the interface (D). Structures were classified in panels A-D according to the outcome of their simulations from figure 3A. p-values were calculated using Wilcoxon tests.

**E.** Percentages of heteromers at the end of the simulation for dimers with different ECOD architectures. Architectures are classified according to their dominant secondary structure (alpha helices, beta strands, or mixed).

Boxplots indicate the median (center lines) and interquartile range (hinges). Whiskers extend from the

hinges of each box to the most extreme values that are at most 1.5 times the interquartile range away from the hinges.

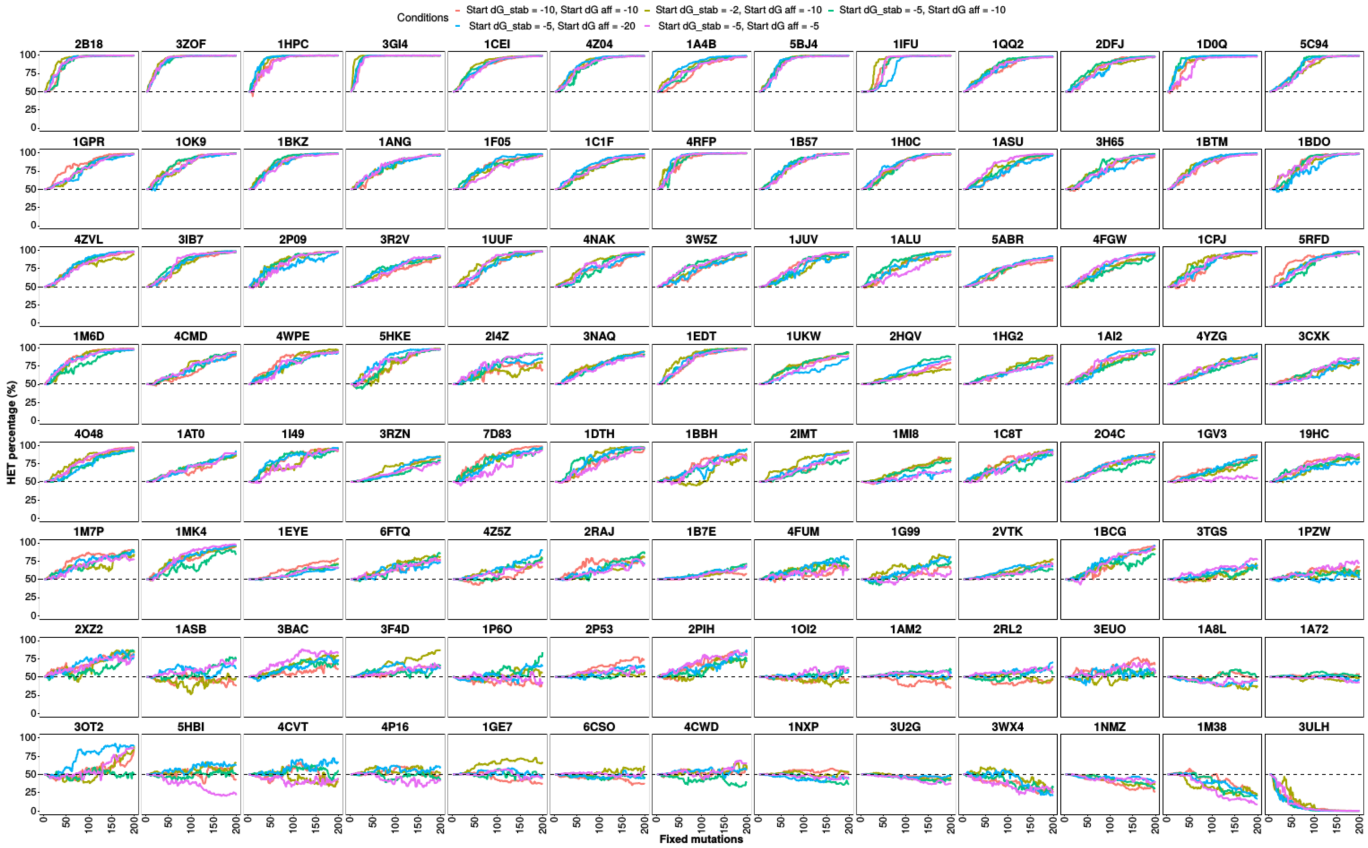


**Appendix Figure S6. Changes in the starting values for $\Delta G_{fold}$ and $\Delta G_{bind}$ do not alter the outcome significantly.** Simulations were run using the distributions of mutational effects for each PDB structure and changing the starting parameters for $\Delta G_{fold}$ and $\Delta G_{bind}$. Each combination of values was selected by doubling or halving one of the reference parameters ($\Delta G_{fold}$ = -5 kcal/mol, $\Delta G_{bind}$ = -10 kcal/mol) while keeping the other one constant.

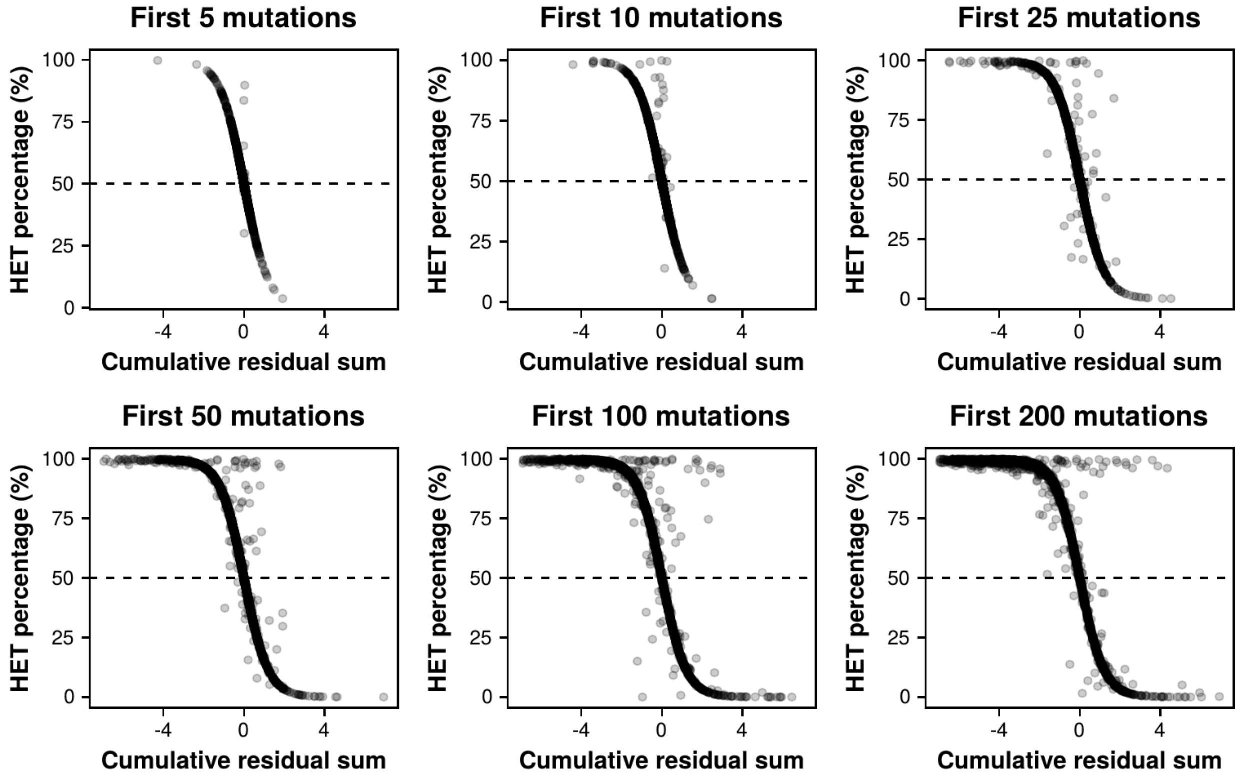


**Appendix Figure S7. The cumulative sum of residuals of fixed mutations reflects the percentage of heteromers.** For each replicate of simulations with each structure, the residuals of fixed mutations up to specific points in the simulation (5, 10, 25, 50, 100, 200 mutations) were added and compared to the percentage of heterodimers at that point in the simulation.

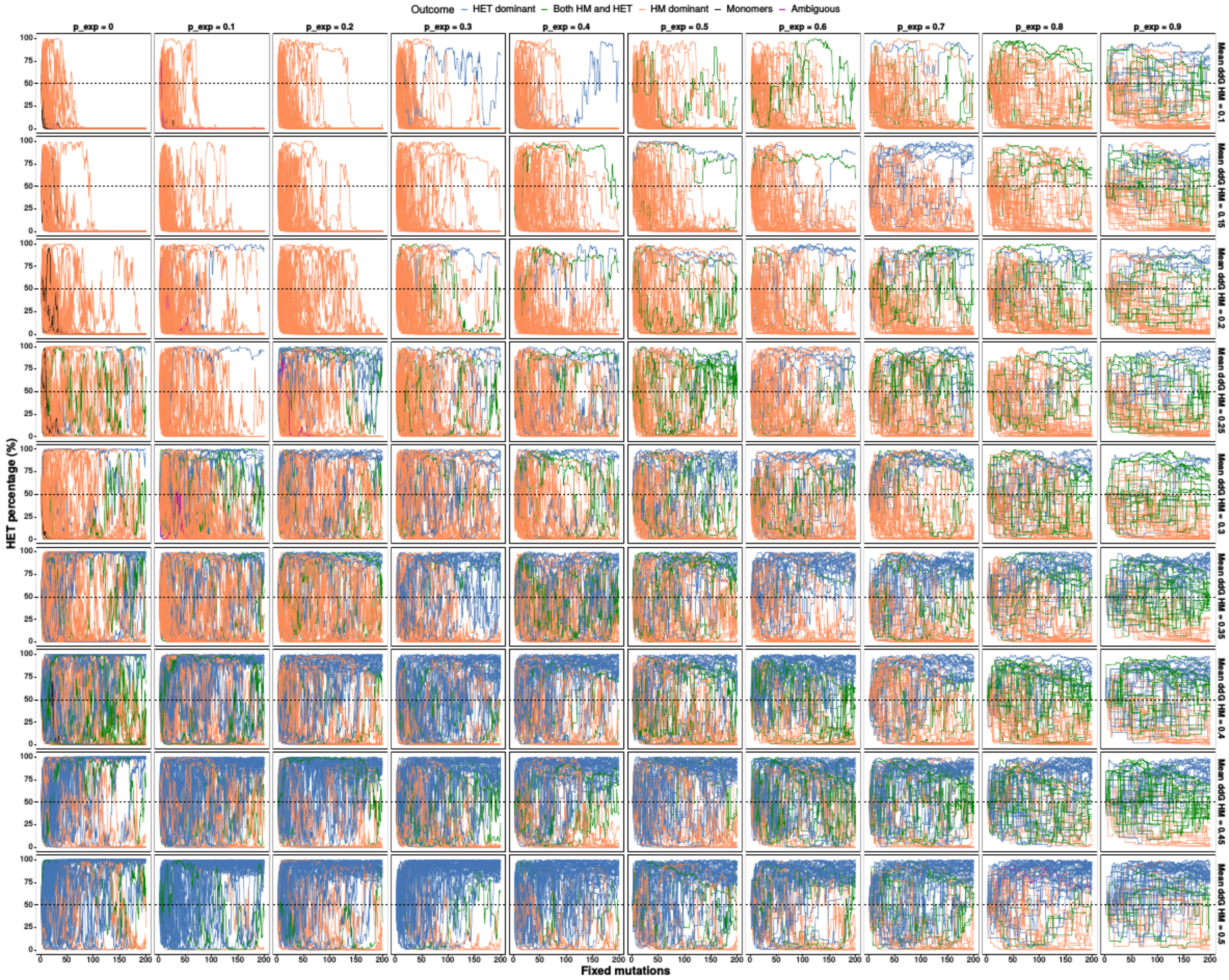


**Appendix Figure S8. Trajectories of individual replicates for parametric simulations that allow changes in synthesis rates.** Individual replicates were colored based on the percentage of heteromers at the end of the simulation following the classification outlined in section 4 of the methods: HET dominant: 70 <= pAB, HM dominant: 70 <= (pAA + pBB), both HM and HET: 70 <= (pAB + pAA + pBB) AND 70 >= pAB AND 70 >= (pAA + pBB), monomers: 70 <= (pA + pB), ambiguous: 70 >= (pAB + pAA + pBB) AND 70 >= (pA + pB). The data shown are the same as in figure 4E.

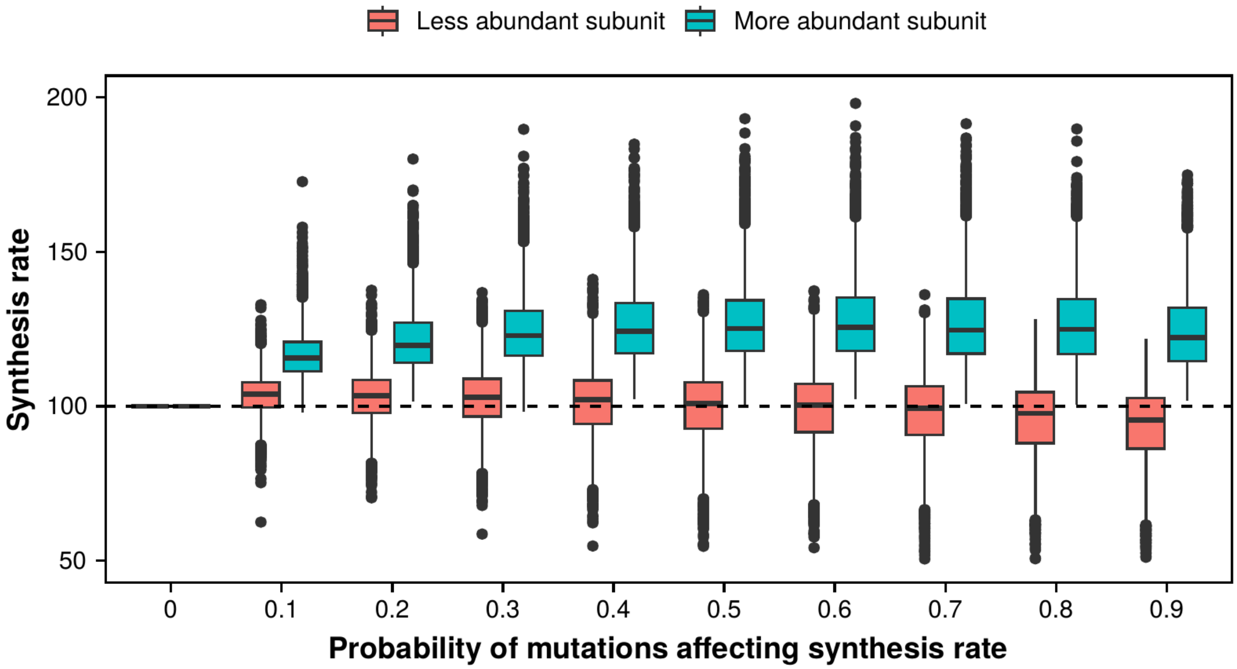


**Appendix Figure S9. Divergence in synthesis rates between paralogs in simulations with structures.** Simulations were run using the distributions of mutational effects for all replicates of each of the 104 PDB structures and different probabilities of mutations affecting synthesis rates. For each simulation, the more abundant subunit and the least abundant subunit were distinguished. The dashed line at 100 indicates the starting synthesis rate for both subunits. Boxplots indicate the median (center lines) and interquartile range (hinges). Whiskers extend from the hinges of each box to the most extreme values that are at most 1.5 times the interquartile range away from the hinges.

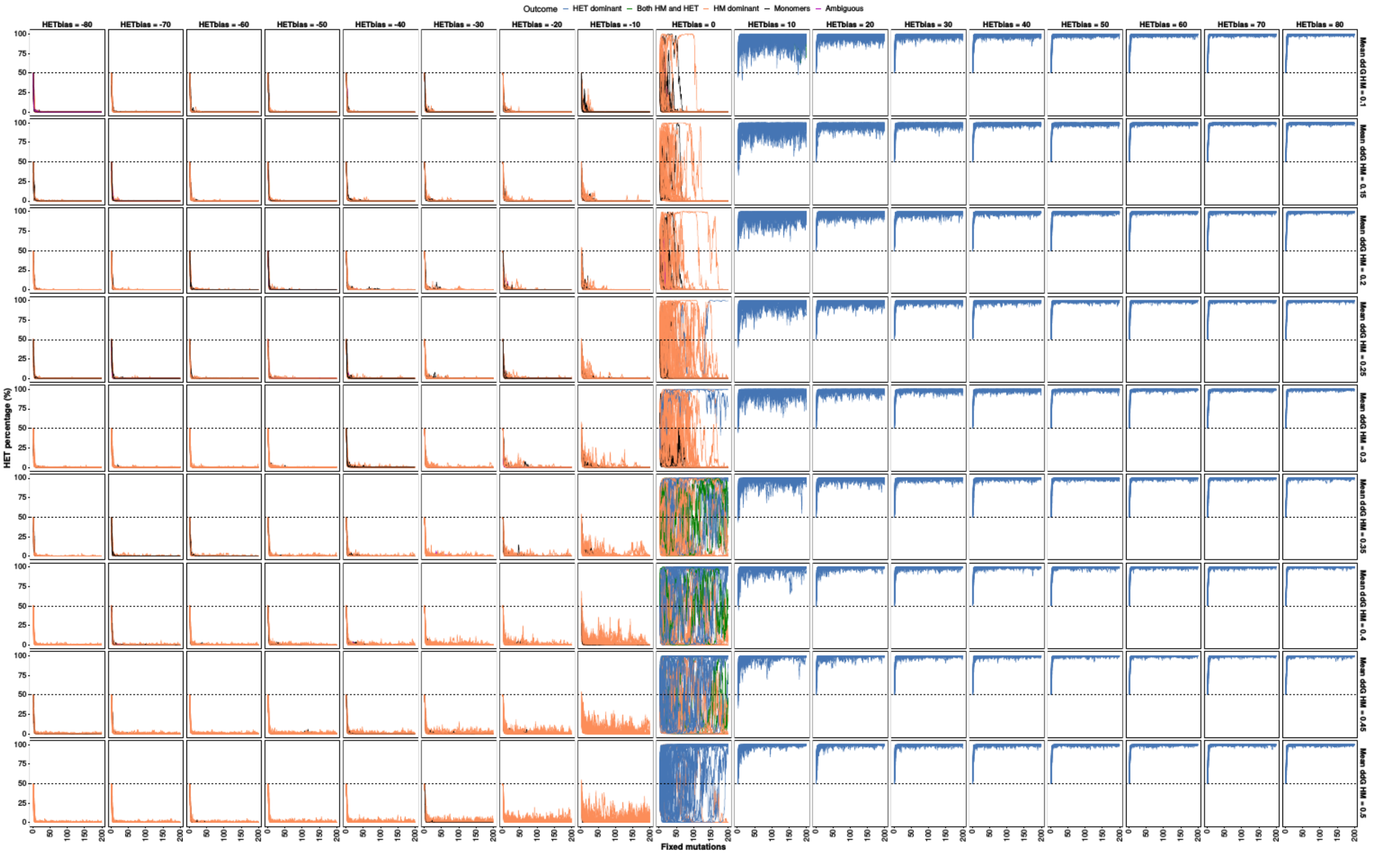


**Appendix Figure S10. Individual trajectories for parametric simulations with differences in specific activities.** Individual replicates were colored based on the percentage of heteromers at the end of the simulation following the classification outlined in section 4 of the methods: HET dominant: 70 <= pAB, HM dominant: 70 <= (pAA + pBB), both HM and HET: 70 <= (pAB + pAA + pBB) AND 70 >= pAB AND 70 >= (pAA + pBB), monomers: 70 <= (pA + pB), ambiguous: 70 >= (pAB + pAA + pBB) AND 70 >= (pA + pB). The data shown are the same as in figure 5E.
