# Supplementary material for: Mutational biases favor complexity increases in protein interaction networks after gene duplication
Source: Mol Syst Biol. 2024 Mar 18;20(5):549–72. doi: 10.1038/s44320-024-00030-z (PMC11066126; doi:10.1038/s44320-024-00030-z)
Supplement: Supplementary file 5 — Expanded View Figures [file 44320_2024_30_MOESM5_ESM.pdf]

Expanded View Figures

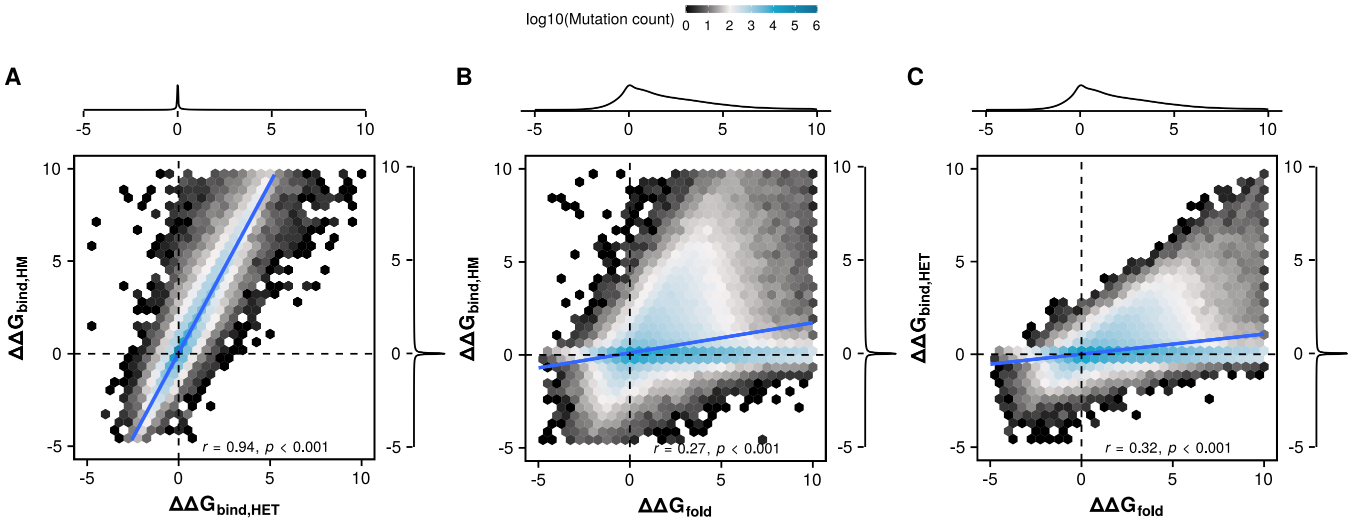

Figure EV1. Bidimensional distributions of mutational effects on $\Delta G_{bind,HET}$, $\Delta G_{bind,HM}$, and ΔG_{fold}.

(**A–C**) Hexagonal bins indicate the density of mutational effects for the pool of mutations for all structures. Two variables and their correlations are shown in each panel: $\Delta G_{bind,HET}$ and $\Delta G_{bind,HM}$ (**A**), $\Delta G_{bind,HM}$ and ΔG_{fold} (**B**), $\Delta G_{bind,HET}$ and ΔG_{fold} (**C**). *P* values are calculated using an asymptotic confidence interval based on Fisher's Z transform for Pearson correlation coefficients.

 © The Author(s)

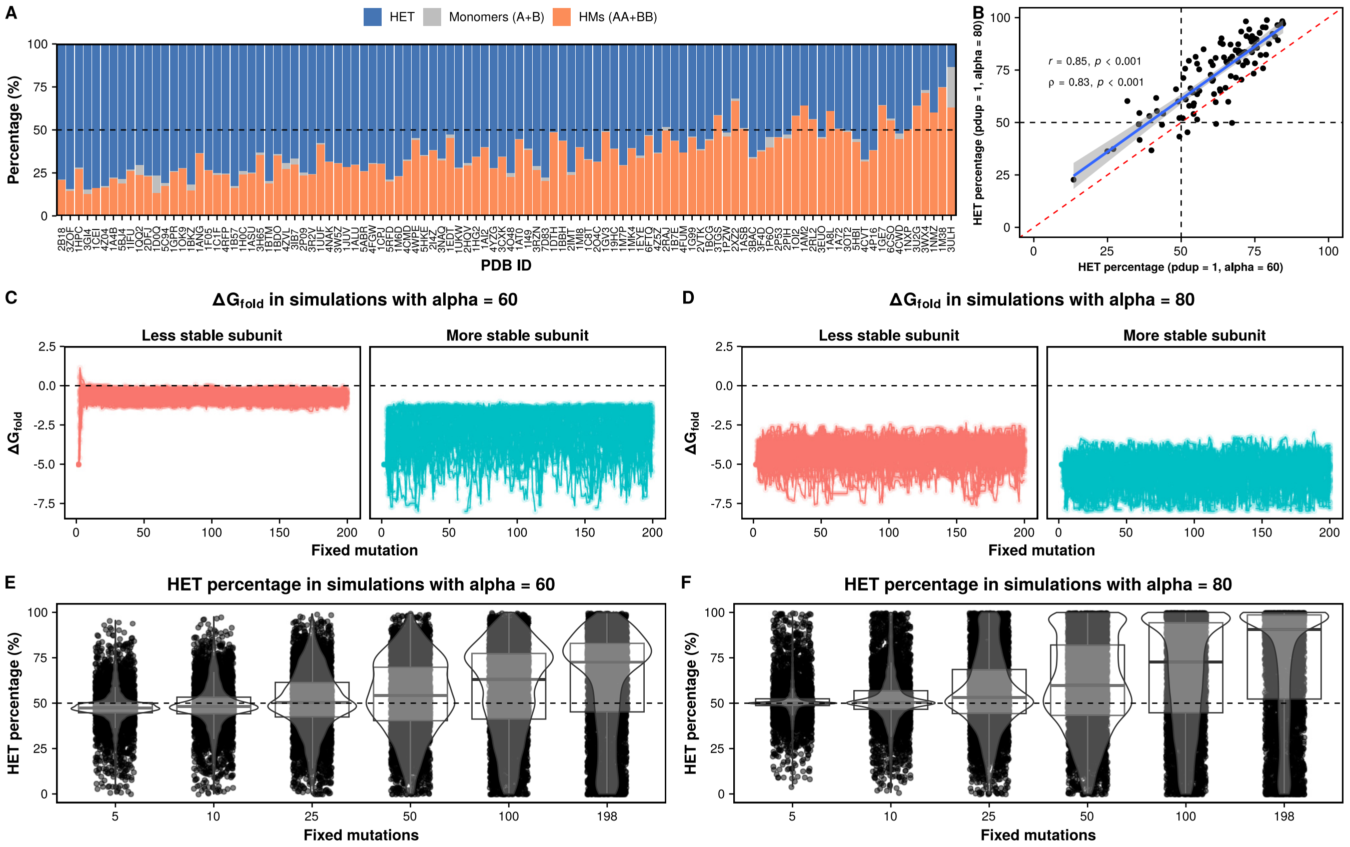

Figure EV2. The general bias observed for heterodimers is also observed when the optimal activity is set to a slightly lower value (alpha = 60).

(**A**) Percentage of heterodimers, homomers, and monomers at the end of simulations with distributions of mutational effects structures when the optimal activity (alpha) is set to 60. Structures are listed in the same order as in Fig. 2C. (**B**) Correlation between the percentages of heterodimers observed at the end of simulations with alpha = 60 (data from Fig. S6A) and simulations with alpha = 80 (data from Fig. 2C). The *P* value for the Spearman correlation coefficient in panel B is calculated using the asymptotic *t* approximation method. The P value for the Pearson correlation coefficient is calculated using an asymptotic confidence interval based on Fisher's Z transform. (**C, D**) Evolution of ΔG_{fold} values in simulations with alpha = 60 (**C**) and alpha = 80 (**D**). For simplicity, only the data for PDB: 1GPR are shown. (**E, F**) Evolution of the percentage of heteromers in simulations with alpha = 60 (**E**) and alpha = 80 (**F**). Replicates for all simulations with all structures are shown. Boxplots in (**E, F**) indicate the median (center lines) and interquartile range (hinges). Whiskers extend from the hinges of each box to the most extreme values that are at most 1.5 times the interquartile range away from the hinges.

© The Author(s)

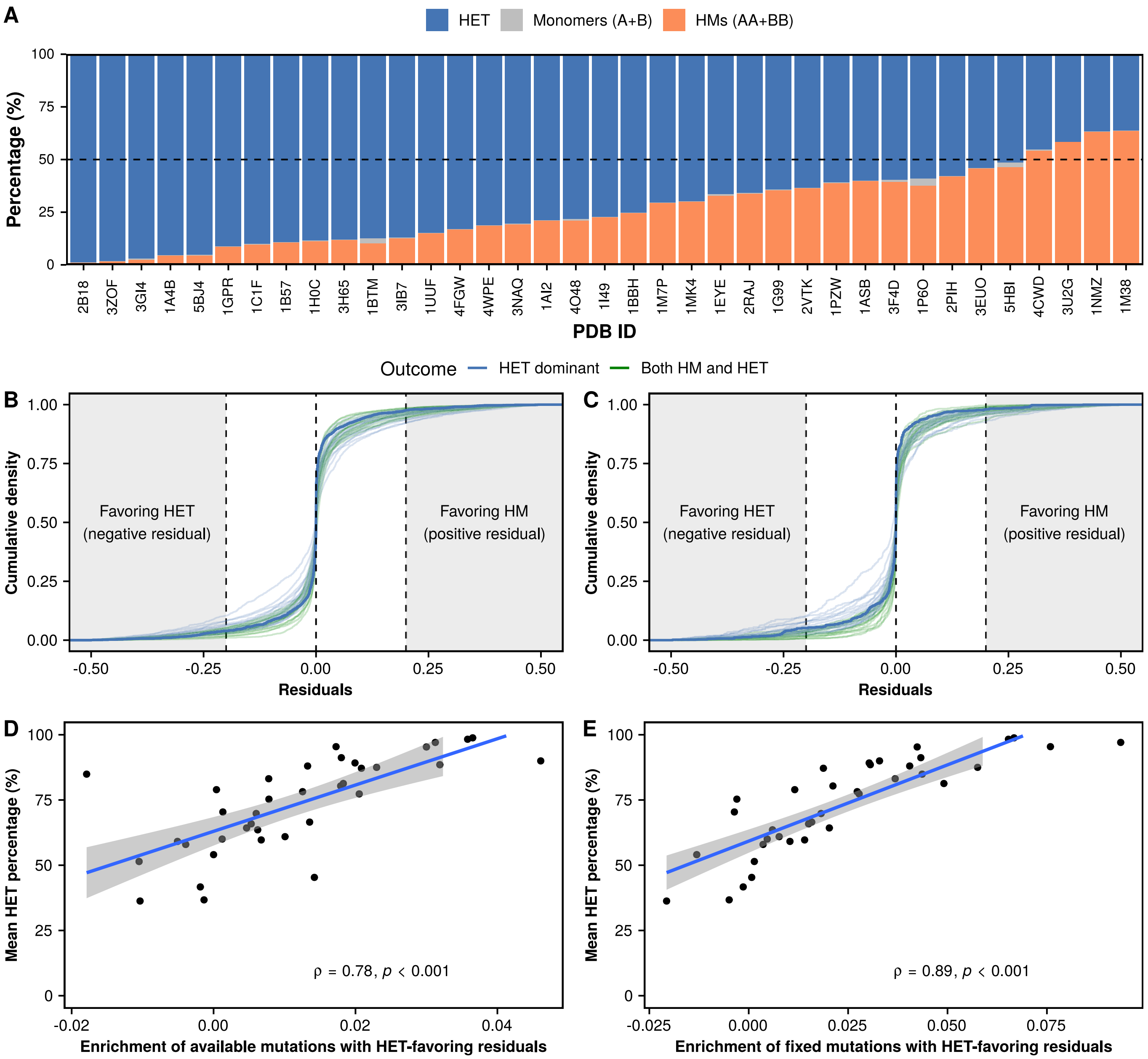

Figure EV3. Mutational biases promote the enrichment of heterodimers in very high quality PDB structures.

(**A**) Average percentages of heterodimers, homodimers, and monomers for each of the very high quality PDB structures at the end of the simulations. The dashed line indicates the starting point at 50% heterodimers and 50% homodimers (25% of each homodimer). (**B, C**) Distributions of available (**B**) and fixed (**C**) residuals with respect to the expectation for each of the 37 very high quality structures. Colors indicate mean outcomes for each structure: HET dominant (heterodimer >70% of dimers), HM dominant (homodimer >70% of dimers), both HM and HET (rest of cases). The thicker blue line represents 2B18. Shaded regions on the left and right indicate mutations whose residuals have a magnitude of at least 0.2 favoring either the heterodimer (negative values) or the homodimer (positive values). (**D, E**) Correlation between enrichment of available (**D**) and fixed (**E**) mutations with residuals favoring the heterodimer and the mean percentage of heterodimers at the end of the simulations. The enrichment of heterodimer-favoring residuals is calculated as the density of mutations with residuals smaller than −0.2 minus the density of mutations with residuals greater than 0.2. *P* values for (**D, E**) are calculated using the asymptotic t approximation method for Spearman correlation coefficients.

© The Author(s)

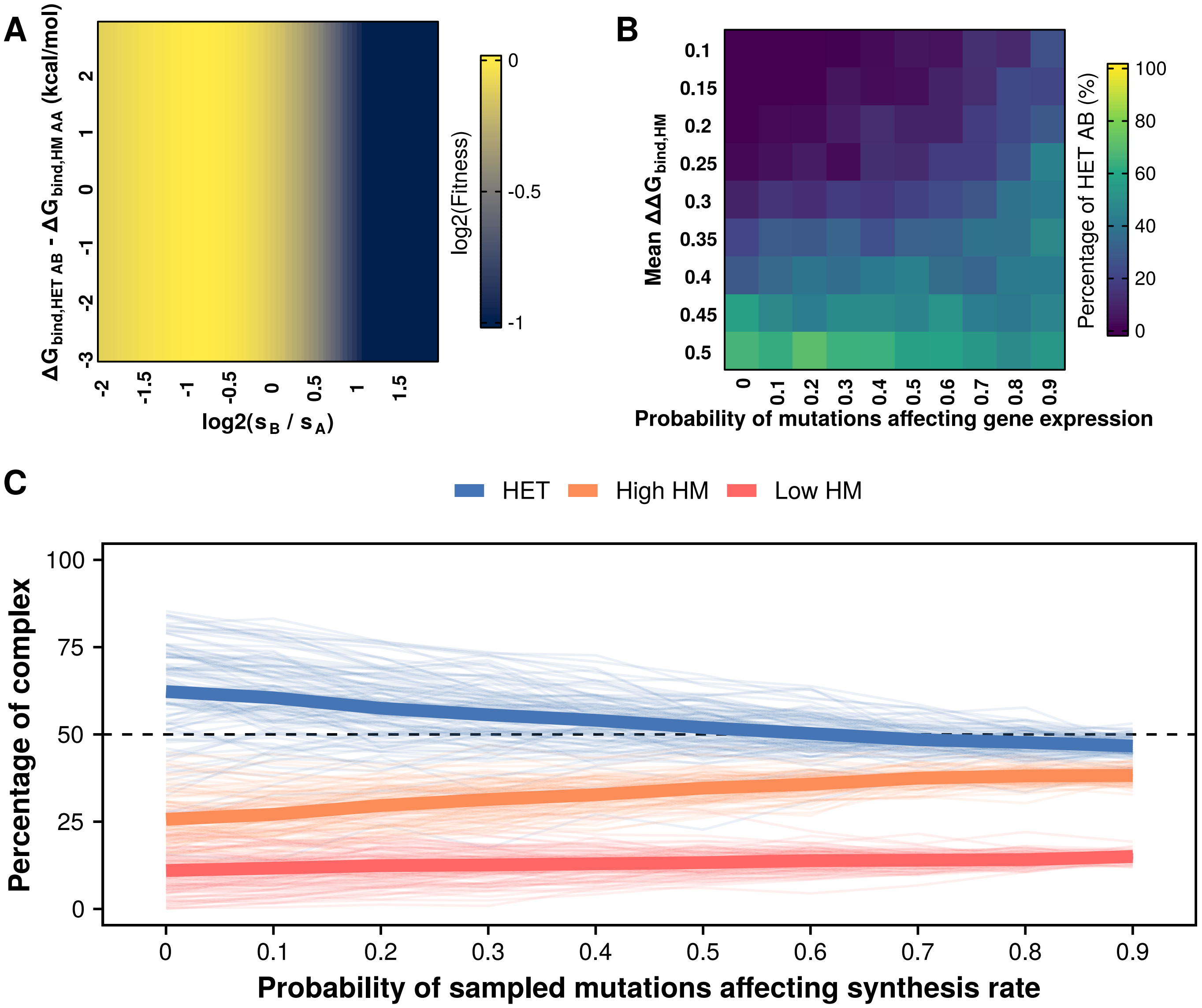

Figure EV4. Simulations allowing changes in synthesis rates with lower optimal activity (alpha = 60).

(**A**) Fitness landscape when the optimal activity is set to 60. Note that s_A, $\Delta\Delta G_{bind,AA}$, and $\Delta\Delta G_{bind,BB}$ remain constant throughout. (**B**) Percentages of heterodimers at the end of parametric simulations allowing changes in synthesis rates and varying mean effects of mutations on homodimers. (**C**) Percentages of heterodimers and each of the two homodimers for each of the 104 tested structures when simulations consider different probabilities of mutations affecting synthesis rates. The two homodimers were distinguished based on their abundance.

© The Author(s)

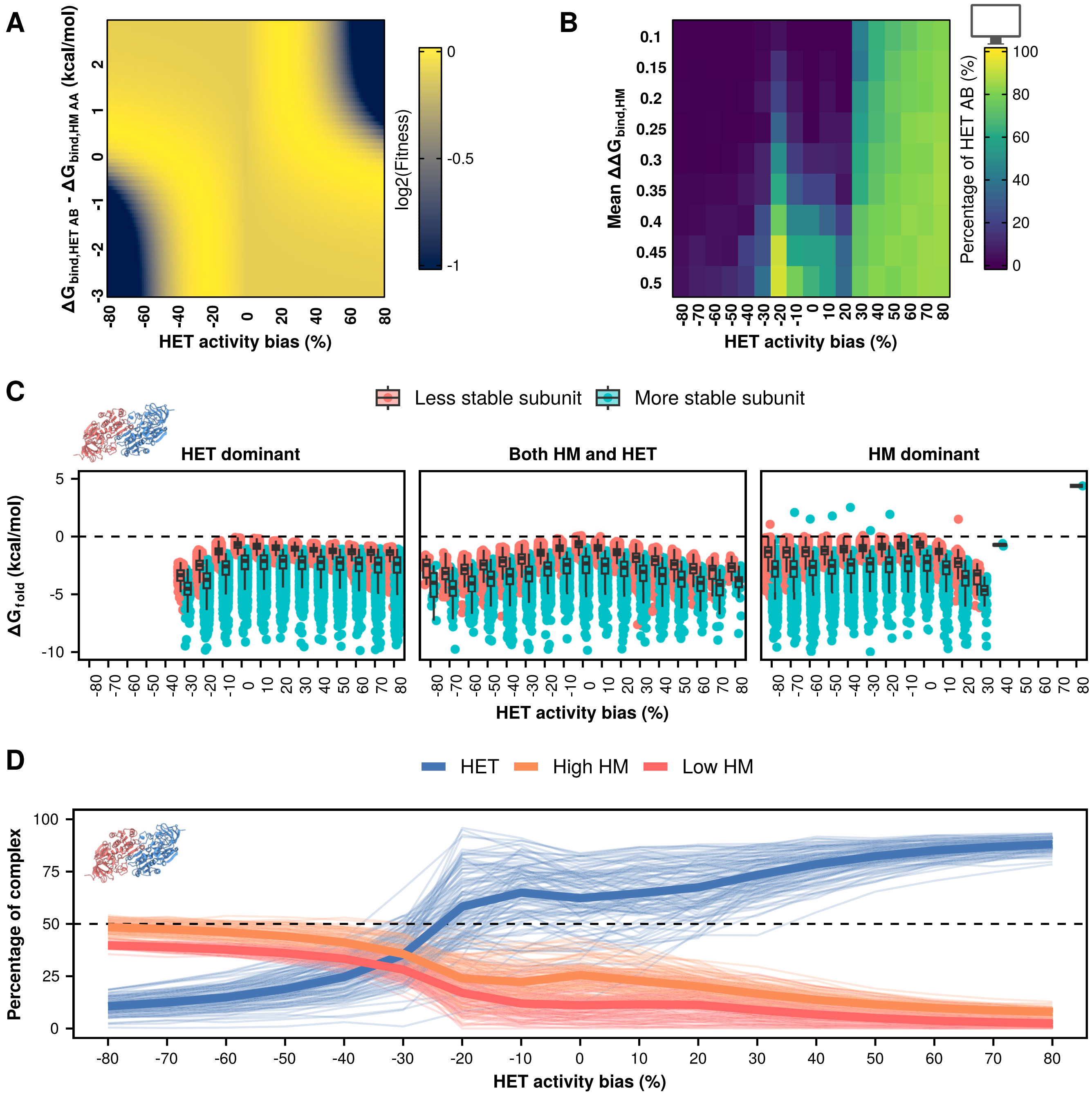

Figure EV5. Simulations with differences in specific activity and lower optimal total activity (alpha = 60).

(**A**) Fitness landscape when the optimal activity is set to 60. $\Delta\Delta G_{bind,AA}$, and $\Delta\Delta G_{bind,BB}$ remain constant throughout at −10 kcal/mol. (**B**) Percentages of heterodimers at the end of parametric simulations with different values of specific activity and varying mean effects of mutations on homodimers. (**C**) ΔG_{fold} values at the end of simulations with alpha = 60 and different specific activities for homo- and heterodimers. Disfavored outcomes, such as HET dominant when the heterodimer is less active, can still be reached if the subunits remain stable. Boxplots in (**C**) indicate the median (center lines) and interquartile range (hinges). Whiskers extend from the hinges of each box to the most extreme values that are at most 1.5 times the interquartile range away from the hinges. (**D**) Percentages of heterodimers and each of the two homodimers for each of the 104 tested structures when simulations consider different specific activities of homo- and heterodimers. The two homodimers were distinguished based on their abundance.

 © The Author(s)
